# Clinical Grade “SNaPshot” Genetic Mutation Profiling in Multiple Myeloma

**DOI:** 10.1016/j.ebiom.2014.11.008

**Published:** 2014-11-18

**Authors:** Elizabeth O'Donnell, Anuj Mahindra, Andrew J. Yee, Valentina Nardi, Nicole Birrer, Nora Horick, Darrell Borger, Dianne Finkelstein, John A. Iafrate, Noopur Raje

**Affiliations:** Massachusetts General Hospital Cancer Center, Boston, MA, United States; Harvard Medical School, Boston, MA, United States

**Keywords:** Myeloma, Mutations, Clinical, Therapy, Sequencing

## Abstract

Whole genome sequencing studies have identified several oncogenic mutations in multiple myeloma (MM). As MM progresses, it evolves genetically underscoring the need to have tools for rapid detection of targetable mutations to optimize individualized treatment. Massachusetts General Hospital (MGH) has developed a Clinical Laboratory Improvement Amendments (CLIA)-approved, high-throughput, genotyping platform to determine the mutation status of a panel of known oncogenes. Sequence analysis using SNaPshot on DNA extracted from bone marrow and extramedullary plasmacytomas is feasible and leads to the detection of potentially druggable mutations. Screening MM patients for somatic mutations in oncogenes may provide novel targets leading to additional therapies for this patient population.

## Highlights

• SNaPshot on deoxyribonucleic acid (DNA) extracted from bone marrow and extramedullary plasmacytomas is feasible.
• SNaPshot can detect potentially druggable mutations and thereby integrate rapid genomic analysis into clinical practice.
• SNaPshot can rapidly identify 152 mutations across 15 genes.

## 1 Introduction

Over the last 10 years, the availability of effective new drugs with acceptable toxicity, such as the immunomodulatory drugs (IMiDs) and the proteasome inhibitors have modified the traditional treatment paradigms in patients with multiple myeloma (MM) with an improvement in the quality and the duration of life (Dimopoulos et al., 2007, Richardson et al., 2014, Jagannath et al., 2004, Richardson et al., 2003, Richardson et al., 2005). Despite these advances, these therapies are non-specific with pleiotropic mechanisms of action that do not target specific genetic mutations. This is due, in part, to the complex genetic architecture of the disease that has, thus far, not been amenable to targeted approaches.

The molecular pathogenesis of MM is complex and incompletely understood. While it is known that there are characteristic chromosomal translocations resulting in over-expression of genes by juxtaposition to the immunoglobulin heavy chain (Ig) locus, these abnormalities cannot fully account for the malignant transformation to MM as many are found in the pre-malignant disease state, monoclonal gammopathy of undetermined significance (MGUS). Mutations and deregulation can occur through diverse pathways. Translocations that place oncogenes under the strong enhancers of the IgH loci, lead to deregulation of the G1 to S transition. Gains and losses of deoxyribonucelic acid (DNA) cause copy number alterations that lead to loss of cell cycle regulators (Morgan et al., 2012). Hyperploidy is associated with increased gene expression and with activating mutations in driver oncogenes. Finally, genetic mutations may also drive deregulation. Though there are few recurrently mutated genes, several of those identified fall within common pathways. Specifically, mutations in *NRAS*, *KRAS* and *BRAF* fall within the extracellular signal-regulated kinase (ERK) pathway (Morgan et al., 2012).

In 2011, Chapman and colleagues published the results of genome sequencing for 38 patients with MM (Chapman et al., 2011). In nearly 50% of patients, mutations were found in genes involving ribonucleic acid (RNA) processing, protein translation and the unfolded protein response. Notably, 16 out of 38 patients had mutations affecting protein translocation and homeostasis highlighting these as therapeutic targets. Statistically significant protein-coding mutations identified included *NRAS*, *KRAS*, *FAM46C*, *DIS3*, *TP53*, *CCND1*, *PNRC1*, *ALOX12B*, *HLA-A*, and *MAGED1*. In addition, one patient had a *BRAF* kinase mutation (G469A) prompting genotyping of an additional 161 MM samples for the 12 most common *BRAF* mutations. Seven additional patients were identified with *BRAF* mutations (4%), a druggable target. More recently, Lohr and colleagues performed parallel sequencing of paired and normal samples from 203 MM patients. Similar to Chapman, frequent mutations were observed in *KRAS*, *NRAS*, *BRAF*, *FAM46C*, *TP53*, and *DIS3* (Lohr et al., 2014). Tumors demonstrated significant heterogeneity with the presence of mutations often in subclonal populations and multiple mutations within the same pathway within the same patient.

MM evolves and invariably progresses through therapy. Bolli and colleagues recently published results of whole-exome sequencing, copy-number profiling, and cytogenetics of myeloma samples, including serial samples in 15 patients, which demonstrated complex clonal evolution over time (Bolli et al., 2014). Moreover, they demonstrated clonal and subclonal heterogeneity within the same patient without the predominant clone necessarily translating at the messenger RNA (mRNA) level. These and other studies demonstrate the complexity of the MM genome underscoring a need for rapid identification of possible druggable oncogenic mutations in order to customize therapy for MM patients.

We have developed a Clinical Laboratory Improvement Amendments (CLIA)-approved, high-throughput, genotyping platform to determine the mutation status of a large panel of known cancer genes. The mutation detection protocol, SNaPshot, uses a highly sensitive multiplexed polymerase chain reaction (PCR)-based assay to simultaneously identify 70 genetic loci frequently mutated in 15 cancer genes. This assay has been used at our institution for over 4 years for tumor genotyping and to help guide therapeutic decisions for patients with various malignancies. We performed SNaPshot analysis on 67 bone marrow samples from MM patients after isolating genomic DNA from these samples after performing quality assurance tests.

## 2 Methods

### 2.1 Patients and Samples

Patients diagnosed with MM and treated at the MGH Cancer Center between 2011 and 2013 who had SNaPshot testing were identified. All subjects voluntarily signed informed consent approved by the institutional review board for SNaPshot testing.

### 2.2 Genotype Analysis

Nucleic acids were extracted from bone marrow samples obtained from myeloma patients using the automated QIAcube system (Qiagen) without any plasma cells. We required 10% of the cells to be malignant MM cells for SNaPshot testing, given the assay sensitivity of overall 5%, as if all 10% clonal plasma cells were carrying the mutation, the mutated allele fraction would be exactly 5%. Multiplexed mutational analysis queried for 152 hotspot mutations distributed across 15 cancer genes, including *AKT1*, *APC*, *BRAF*, *CTNNB1*, *EGFR*, *FLT3*, *ERBB2*, *IDH1*, *IDH2*, *JAK2*, *KIT*, *KRAS*, *MAP2K1*, *NOTCH1*, *NRAS*, *PIK3CA*, *PTEN*, and *TP53* (Supp. Table 1) was performed using a custom modified ABI PRISM® SNaPshot™ Multiplex System on an ABI PRISM 3730 DNA Analyzer, as previously described (Dias-Santagata et al., 2010, Dias-Santagata et al., 2011). The data were interpreted with GeneMapper Analysis Software (Life Technologies/Applied Biosystems). Testing of the tumor suppressor genes *TP53*, *APC* and *PTEN* was limited to common mutation sites, covering 29%, 15% and 15%, respectively, of all known somatic mutations in these genes. Patients were classified according to whether any of the 15 tested genes were mutated versus wild type.

### 2.3 Statistical Analysis

The distributions of clinical variables in patients with wild type versus mutated tumors were compared using Fisher's exact test (categorical variables) and Wilcoxon rank sum test (continuous variables). Median survival for the whole group and by mutation status (mutation vs. wild type) was calculated using the Kaplan–Meier method and the log-rank test was used to compare survival by mutation status.

## 3 Results

The majority (55/67) of samples taken were at time of relapse. However, it is noteworthy that 3/12 specimens obtained in the upfront setting did have oncogenic mutations. When comparing samples of patients with mutations versus those without, there were no significant differences between stage at diagnosis, age, and type of heavy chain monoclonal protein (Table 1). The majority of mutations were observed in patients with heavy chain disease (24/26). Patients with mutations had a median of 4 prior lines of therapy versus 2 in wild type patients (*p* = 0.03). When baseline cytogenetics were compared between relapsed patients with mutations versus wild type, no significant differences were appreciated.

Thirty-nine % (26/67) of tumor samples from 67 unique patients harbored candidate mutations. 3/26 were BM samples from newly diagnosed patients and 23/26 were bone marrow (22/26) and plasmacytoma (1/26) samples from relapsed MM. All samples with mutations had single mutations. Somatic oncogenic mutations were found in *KRAS* (15), *NRAS* (6), *BRAF* (2), *TP53* (2), and *HRAS* (1) (Table 2).

## 4 Discussion

Clinical grade sequence analysis using SNaPshot on DNA extracted from bone marrow and extramedullary plasmacytomas is feasible and leads to the detection of potentially druggable mutations. Mutations were found in 39% (26/67) of patients in our 67-patient cohort. Although whole exome or whole genome sequencing is possible in the research laboratory, the routine use of these methods in clinical practice is time and cost-limited. SNaPshot provides a rapid, reasonable-cost method of identifying oncogenic mutations in MM patient samples. A limitation of this technology is that only pre-identified mutations are located and that other mutations not included in SNaPshot could potentially be missed. This likely resulted in a lower incidence of mutations in our patient population. Additionally, our assay was performed on unpurified MM cells and this may contribute to a higher false negative rate in our assay and is a limitation of the study. This is in contrast with a new SNaPshot NGS assay that the Center for Integrated Diagnostic has developed at our institution which is a targeted next generation sequencing assay and is not amplicon based. Our next generation test, the SNaPshot NGS assay, fully sequences 1000 cancer genes. This assay utilizes a multiplex PCR technology called Anchored Multiplex PCR for single nucleotide variant and insertion/deletion (indel) detection in genomic DNA using next generation sequencing. This assay has actually similar sensitivity to the SNaPshot multiplexed amplicon based assay (5%), but is quantitative and therefore allows for the calculation of the mutational load. Furthermore, the new assay although targeted, sequences entire exons and not just a few hotspot nucleotides, allowing for detection of additional mutations. One of the strengths of having an internal testing platform is the ability to update our panel as needed as new critical targets emerge. Costlier and lengthier commercially available testing does not necessarily provide therapeutic guidance. By focusing our analysis to these most relevant targets, we can significantly reduce costs and turnaround time, as well as provide results to clinicians that offer the clearest implications for best treatment options for that patient.

The understanding of the clinical implications of somatic mutations in MM is an evolving field. Several mutated oncogenes were found in our cohort of MM specimens including *KRAS*, *NRAS*, *BRAF*, and *TP53*. In the era of personalized medicine, the availability of rapid, high throughput genomic sequencing opens up the possibility of efficiently integrating genomic analysis into our clinical practice. Case reports of success with targeted therapy for BRAF mutations in MM provide a foundation of optimism in support of further investigation (O'Donnell & Raje, 2013). There is currently an open-label, phase II study of vemurafenib in patients with BRAF V600 mutation-positive cancers which includes myeloma patients (NCT01524978). Mulligan and colleagues recently reported that NRAS mutations were associated with lower response rates to single-agent bortezomib but not to high dose dexamethasone, an association that was not found in patients with KRAS mutations. Further work is needed to understand if the impact of NRAS is drug-specific but their work does highlights the importance of understanding the relationship of mutations with response to therapy (Mulligan et al., 2014). However, as Lohr and colleagues discuss, the clinical benefit of targeted therapies in a tumor defined by subclonality may prove challenging (Lohr et al., 2014). Effective targeted therapy may require a better understanding of the extent of clonal heterogeneity. Having a technology that can facilitate this understanding may prove valuable in the further development of effective targeted therapies. Future strategies of treatment may include the combination of targeted therapies with existing proteosome inhibitors and immunomodulatory drugs. This analysis highlights the feasibility of integrating rapid genomic analysis into clinical practice.

The following are the supplementary data related to this article.

Supplemental Table 1 Cancer gene mutations evaluated by the SNaPshot genotyping assay.

## Disclosure of Potential Conflicts of Interest

NSR is a consultant for Celgene, Millennium, Onyx, Amgen and Novartis. She has research funding from the Eli Lilly, Acetylon and Onyx.

## Financial Support

NSR was funded by the LLS Clinical Scholar Award (2194-10). The funding body had no role in the study design, data collection, data analysis, data interpretation or writing of the manuscript.

## Figures and Tables

**Table 1:** Patient characteristics.

Characteristics	Wild type (n = 41)	Mutated (n = 26)	P value
ISS stage at diagnosis, n (%)			
I	15 (36.6)	9 (34.6)	1.00
II	11 (26.8)	6 (23.1)	
III	14 (34.1)	9 (34.6)	
Unknown	1 (2.4)	2 (7.7)	
Median age at Dx, years (range)	64 (38–83)	60 (47–74)	0.46
M-protein, n (%)			
IgG K	11 (26.8)	9 (34.6)	0.15
IgG L	6 (14.6)	6 (23.1)	
IgA K	6 (14.6)	4 (15.4)	
IgA L	3 (7.3)	5 (19.2)	
K light	9 (22.0)	1 (3.8)	
L light	6 (14.6)	1 (3.8)	
Heavy chain	26 (63.4)	24 (92.3)	0.009
Light chain	15 (36.6)	2 (7.7)	
Timing of SNaPshot, n (%)			
Upfront	9 (22.0)	3 (11.5)	0.34
Relapse	32 (78.0)	23 (88.5)	
Med. no of prior Tx (range)	2 (1–7)	4 (1–7)	0.07
ASCT, n (%)	18 (56.3)	15 (65.2)	0.58
Lenalidomide, n (%)	29 (90.6)	22 (95.7)	0.63
Bortezomib, n (%)	29 (90.6)	22 (95.7)	0.63
Cyclophosphamide, n (%)	18 (56.3)	20 (87.0)	0.02

ASCT — autologous stem cell transplant.

**Table 2:** Mutations detected by SNaPshot.

Mutation	n = 67
KRAS (n = 15)	15 (22.4)
c.183A > C (p.Q61H)	4
c.35G > A (p.G12D)	3
c.34G > C (p.G12R)	2
c.34G > T (p.G12C)	2
c.35G > C (p.G12A)	2
c.34G > A (p.G12S)	1
c.35G > T (p.G12V)	1
NRAS (n = 6)	6 (8.9)
c.38G > A (p.G13D)	2
c.183A > C (p.Q61H)	2
c.182A > G (p.Q61R)	1
c.35G > A (p.G12N)	1
HRAS (n = 1)	1 (1.5)
c.181 C > A (p.Q61K)	1
BRAF (n = 2)	2 (3)
c.1799 T > A (p.V600E)	1
c.1406G > A (p.G469E)	1
p53 (n = 2)	2 (3)
c.743G > A (p.R248G)	1
c.818G > A (p.R273H)	1
Wild type	41 (61.2)
