# Supplementary material for: Clinical Grade “SNaPshot” Genetic Mutation Profiling in Multiple Myeloma
Source: eBioMedicine. 2014 Nov 18;2(1):71–3. doi: 10.1016/j.ebiom.2014.11.008 (PMC4485483; doi:10.1016/j.ebiom.2014.11.008)
Supplement: Supplemental Table 1 — Cancer gene mutations evaluated by the SNaPshot genotyping assay. [file mmc1.pdf]

Supplemental Table 1: Cancer Gene Mutations Evaluated by the SNaPshot Genotyping Assay

Gene	DNA	AA
AKT1	c.49G>A	p.Glu17Lys
APC	c.4666_4667insA	p.Thr1556fs*3
	c.3340C>T	p.Arg1114X
	c.4012C>T	p.Gln1338X
	c.4348C>T	p.Arg1450X
BRAF	c.1397G>A	p.Gly466Glu
	c.1397G>C	p.Gly466Ala
	c.1397G>T	p.Gly466Val
	c.1406G>A	p.Gly469Glu
	c.1406G>C	p.Gly469Ala
	c.1406G>T	p.Gly469Val
	c.1789C>G	p.Leu597Val
	c.1798_1799GT>AA	p.Val600Lys
	c.1798G>A	p.Val600Met
	c.1798G>T	p.Val600Leu
	c.1799T>A	p.Val600Glu
	c.1799T>C	p.Val600Ala
	c.1799T>G	p.Val600Gly
CTNNB1	c.101G>A	p.Gly34Glu
	c.101G>T	p.Gly34Val
	c.109T>A	p.Ser37Thr
	c.109T>C	p.Ser37Pro
	c.109T>G	p.Ser37Ala
	c.110C>A	p.Ser37Tyr
	c.110C>G	p.Ser37Cys
	c.110C>T	p.Ser37Phe
	c.121A>C	p.Thr41Pro
	c.121A>G	p.Thr41Ala
	c.121A>T	p.Thr41Ser
	c.122C>A	p.Thr41Asn
	c.122C>G	p.Thr41Ser
	c.122C>T	p.Thr41Ile
	c.133T>A	p.Ser45Thr
	c.133T>C	p.Ser45Pro
	c.133T>G	p.Ser45Ala
	c.134C>A	p.Ser45Tyr
	c.134C>G	p.Ser45Cys
	c.134C>T	p.Ser45Phe
	c.94G>A	p.Asp32Asn
	c.94G>C	p.Asp32His

	c.94G>T	p.Asp32Tyr
	c.95A>C	p.Asp32Ala
	c.95A>G	p.Asp32Gly
	c.95A>T	p.Asp32Val
	c.98C>A	p.Ser33Tyr
	c.98C>G	p.Ser33Cys
	c.98C>T	p.Ser33Phe
EGFR	c.2155G>A	p.Gly719Ser
	c.2155G>T	p.Gly719Cys
	c.2156G>A	p.Gly719Asp
	c.2156G>C	p.Gly719Ala
	c.2235_2249del	p.Glu746_Ala750del
	c.2236_2250del	p.Glu746_Ala750del
	c.2369C>T	p.Thr790Met
	c.2573T>G	p.Leu858Arg
	c.2582T>A	p.Leu861Gln
	c.2582T>G	p.Leu861Arg
	Exon 19 insertion/deletion	
	Exon 20 insertion/deletion	
ERBB2	Exon 20 insertion	
IDH1	c.394C>A	p.Arg132Ser
	c.394C>G	p.Arg132Gly
	c.394C>T	p.Arg132Cys
	c.395G>A	p.Arg132His
	c.395G>T	p.Arg132Leu
KIT	c.2447A>C	p.Asp816Ala
	c.2447A>G	p.Asp816Gly
	c.2447A>T	p.Asp816Val
KRAS	c.181C>A	p.Gln61Lys
	c.181C>G	p.Gln61Glu
	c.181C>T	p.Gln61X
	c.182A>C	p.Gln61Pro
	c.182A>G	p.Gln61Arg
	c.182A>T	p.Gln61Leu
	c.183A>C	p.Gln61His
	c.183A>T	p.Gln61His
	c.34G>A	p.Gly12Ser
	c.34G>C	p.Gly12Arg
	c.34G>T	p.Gly12Cys
	c.35G>A	p.Gly12Asp
	c.35G>C	p.Gly12Ala
	c.35G>T	p.Gly12Val
	c.37G>A	p.Gly13Ser

	c.37G>C	p.Gly13Arg
	c.37G>T	p.Gly13Cys
	c.38G>A	p.Gly13Asp
	c.38G>C	p.Gly13Ala
	c.38G>T	p.Gly13Val
MAP2K1	c.167A>C	p.Gln56Pro
	c.171G>C	p.Lys57Asn
	c.171G>T	p.Lys57Asn
	c.199G>A	p.Asp67Asn
NOTCH1	c.4724T>C	p.Leu1575Pro
	c.4802T>C	p.Leu1601Pro
NRAS	c.181C>A	p.Gln61Lys
	c.181C>G	p.Gln61Glu
	c.181C>T	p.Glu61X
	c.182A>C	p.Gln61Pro
	c.182A>G	p.Gln61Arg
	c.182A>T	p.Gln61Leu
	c.183A>C	p.Gln61His
	c.183A>T	p.Gln61His
	c.34G>A	p.Gly12Ser
	c.34G>C	p.Gly12Arg
	c.34G>T	p.Gly12Cys
	c.35G>A	p.Gly12Asp
	c.35G>C	p.Gly12Ala
	c.35G>T	p.Gly12Val
	c.37G>A	p.Gly13Ser
	c.37G>C	p.Gly13Arg
	c.37G>T	p.Gly13Cys
	c.38G>A	p.Gly13Asp
	c.38G>C	p.Gly13Ala
	c.38G>T	p.Gly13Val
PIK3CA	c.1624G>A	p.Glu542Lys
	c.1624G>C	p.Glu542Gln
	c.1633G>A	p.Glu545Lys
	c.1633G>C	p.Glu545Gln
	c.1636C>A	p.Gln546Lys
	c.1636C>G	p.Gln546Glu
	c.1637A>C	p.Gln546Pro
	c.1637A>G	p.Gln546Arg
	c.1637A>T	p.Gln546Leu
	c.263G>A	p.Arg88Gln
	c.3139C>T	p.His1047Tyr
	c.3140A>G	p.His1047Arg

	c.3140A>T	p.His1047Leu
	c.3145G>A	p.Gly1049Ser
	c.3145G>C	p.Gly1049Arg
PTEN	c.799delA	p.Lys267fs*9
	c.800delA	p.Lys267fs*9
	c.388C>G	p.Arg130Gly
	c.388C>T	p.Arg130X
	c.517C>T	p.Arg173Cys
	c.697C>T	p.Arg233X
TP53	c.524G>A	p.Arg175His
	c.524G>T	p.Arg175Leu
	c.733G>A	p.Gly245Ser
	c.733G>C	p.Gly245Arg
	c.733G>T	p.Gly245Cys
	c.742C>G	p.Arg248Gly
	c.742C>T	p.Arg248Trp
	c.743G>A	p.Arg248Gln
	c.743G>C	p.Arg248Pro
	c.743G>T	p.Arg248Leu
	c.817C>A	p.Arg273Ser
	c.817C>G	p.Arg273Gly
	c.817C>T	p.Arg273Cys
	c.818G>A	p.Arg273His
	c.818G>C	p.Arg273Pro
	c.818G>T	p.Arg273Leu
	c.916C>T	p.Arg306X
